# CD8+ T Lymphocyte Expansion, Proliferation and Activation in Dengue Fever

**DOI:** 10.1371/journal.pntd.0003520

**Published:** 2015-02-12

**Authors:** Andréia Manso de Matos, Karina Inacio Carvalho, Daniela Santoro Rosa, Lucy Santos Villas-Boas, Wanessa Cardoso da Silva, Célia Luiza de Lima Rodrigues, Olímpia Massae Nakasone Peel Furtado Oliveira, José Eduardo Levi, Evaldo Stanislau Affonso Araújo, Claudio Sergio Pannuti, Expedito José Albuquerque Luna, Esper George Kallas

**Affiliations:** 1 Universidade de São Paulo, Faculdade de Medicina, Disciplina de Imunologia Clínica e Alergia (LIM-60), São Paulo, Brazil; 2 Albert Einstein Hospital, São Paulo, Brazil; 3 Division of Immunology, Federal University of São Paulo (UNIFESP), Santos, São Paulo, Brazil; 4 Universidade de São Paulo, Instituto de Medicina Tropical de São Paulo e Faculdade de Medicina, Departamento de Moléstias Infecciosas e Parasitárias—(LIM-52), São Paulo, Brazil; 5 Hospital Ana Costa, Santos, São Paulo, Brazil; 6 Universidade de São Paulo, Faculdade de Medicina, Departamento de Moléstias Infecciosas e Parasitárias (LIM-47), São Paulo, Brazil; University of California, Berkeley, UNITED STATES

## Abstract

Dengue fever induces a robust immune response, including massive T cell activation. The level of T cell activation may, however, be associated with more severe disease. In this study, we explored the level of CD8+ T lymphocyte activation in the first six days after onset of symptoms during a DENV2 outbreak in early 2010 on the coast of São Paulo State, Brazil. Using flow cytometry we detected a progressive increase in the percentage of CD8+ T cells in 74 dengue fever cases. Peripheral blood mononuclear cells from 30 cases were thawed and evaluated using expanded phenotyping. The expansion of the CD8+ T cells was coupled with increased Ki67 expression. Cell activation was observed later in the course of disease, as determined by the expression of the activation markers CD38 and HLA-DR. This increased CD8+ T lymphocyte activation was observed in all memory subsets, but was more pronounced in the effector memory subset, as defined by higher CD38 expression. Our results show that most CD8+ T cell subsets are expanded during DENV2 infection and that the effector memory subset is the predominantly affected sub population.

## Author Summary

Dengue is a disease affecting approximately 400 million people annually, especially in tropical and subtropical areas of the globe. The immune response against the dengue virus is still under investigation and it is important to understand why the disease can be fatal in a small proportion of cases. In this work, we explored how an important cell type of the immune system, namely the CD8+ T cell, reacts during dengue infection. Using a method known as flow cytometry, we demonstrated that these cells expand and become highly activated, during the days following the onset of dengue fever symptoms. This expansion is associated with a decreased dengue virus load in the patients’ blood, suggesting that CD8+ T cells play an important role in viral control. Interestingly, we found that a subset of CD8+ T cells, called effector memory, is greatly expanded during dengue infection. Our results are important because they might contribute to the understanding of disease mechanisms during dengue infection and may help in the development of a novel vaccine against dengue.

## Introduction

Dengue is the most prevalent arthropod-born viral disease in tropical and subtropical areas of the globe, affecting approximately 400 million people annually [1]. The World Health Organization estimates that nearly 40% of the world’s population lives in areas at risk for dengue transmission. Dengue cases in Central and Latin America have increased almost five-fold in the last 30 years. During 2008, up to one million cases were reported in Americas, and higher numbers of deaths were documented in the South [2]. In the latest decades, Brazil has been hard hit by the disease, accounting for more than 60% of the total reported cases in the Americas [2]. The continuing occurrence of the disease in resource limited countries and the lack of novel therapeutic approaches or a highly effective vaccine make dengue fever a neglected disease. Surveillance for dengue is absent in most countries, and no existing model for predicting an outbreak in endemic regions is widely available. Therefore, it is important to increase our knowledge of disease pathogenesis, with the goal of developing new strategies to fight the epidemic.

The mechanisms by which the dengue virus (DENV) causes severe illness remain to be elucidated. Both biological properties of the viral isolates and immunogenic host factors seem to contribute to the level of pathogenicity [3,4,5,6]. Whereas immunity induced by natural infection is believed to provide serotype-specific lifelong protection, previous infection by a distinct serotype is considered to increase the risk for the development of dengue hemorrhagic fever (DHF) and dengue shock syndrome (DSS) [5,7]. The immunological processes during dengue infection are not yet completely defined. However, incidence of mild dengue manifestations and occasional progression to the more severe disease likely reflect a complex interplay between host and viral factors including cytokine production by inflammatory cells. Previous studies reported increased levels of circulating cytokines and soluble receptors in DHF patients when compared to those with dengue fever (DF), suggesting that immune activation may be related to disease severity [8]. T cell activation mechanisms are based on the binding of specific T cell receptors (TCRs) to MHC molecules [9]. CD8+ T cells are one of the most important cell types to recognize and eliminate infected cells. Some authors have suggested that high numbers of CD8+ T cells might be protective by reducing viral load [10]. Memory T lymphocytes remain present in the absence of antigenic stimulation and have the capacity to expand rapidly upon secondary challenge. In the last decade, several surface markers have been used to distinguish among effector memory (TEM), central memory (TCM), and terminally differentiated memory cells (TEMRA) [11]. In this work, we explored the state of CD8+ T cell activation in different compartments during the acute phase of dengue fever.

## Methods

### Ethics statement

All procedures adopted in this study were performed according to the terms agreed by the Institutional Review Board from the *Hospital das Clínicas*, University of São Paulo (CAPPesq—Research Projects Ethics Committee). This study was approved by CAPPesq under protocol 0652/09. Written informed consents were obtained from all study volunteers.

### Clinical samples

Whole-blood samples were collected, using sterile EDTA-treated Vacutainer tubes (BD Brazil), from patients with DENV2 dengue at the Ana Costa Hospital, Santos, State of São Paulo, during the 2010 first semester outbreak [12]. Patients with suspected dengue fever, dengue with warning signs or severe dengue were invited to participate in the study. A rapid rest was performed to confirm the diagnosis of acute dengue disease, followed by the detection of dengue viral load determination (see next sections). Primary dengue infection was considered when dengue IgG-specific antibodies were not detected in the presence of reactive dengue IgM-specific antibody and/or NS1 antigenemia. Secondary dengue infection was considered in the presence of dengue IgG-specific antibodies at acute phase up to 6 days of symptoms.

### Serology

A commercial Dengue Duo Test Bioeasy (Standard Diagnostic Inc. 575–34, Korea), a rapid test kit, was used for dengue diagnosis, by detection of both dengue virus NS1 antigen and IgM- and IgG-specific antibodies in human blood. Samples were considered positive for acute dengue fever when NS1 or IgM bands were reactive in the testing kit. We also considered an acute case whenever DENV2 RNA was detected.

### Dengue viral load determination

The IgG avidity test was used to determine if patients presented with a primary or secondary DENV infection [13]. Samples with low avidity IgG antibodies were classified as primary DENV infection, whereas samples with high avidity IgG antibodies were classified as secondary. Samples in which IgG antibodies were not detected could not be classified, although the majority were probably from primary DENV infection. Viral load was determined by an “in-house” real-time polymerase chain reaction (RT-PCR) method. RNA was extracted from 140μL of plasma using the Qiagen Viral RNA kit (Qiagen, USA). All RT-PCR reactions were performed in duplicate. RT-PCR was conducted using the SuperScript III Platinum SYBR Green One-Step qRT-PCR kit with ROX (Invitrogen, USA) and 0.4 μM of primers covering all four DENV serotypes [14]. Cycling conditions were: 10 minutes reverse transcription at 60°C, 1 min Taq polymerase activation at 95°C, followed by 45 cycles consisting of 95°C without holding time, 60°C for 3 seconds, and 72°C for 10 seconds. The reaction was run on an ABI 7300 RT-PCR equipment (Applied Biosystems, Brazil). As an internal control Bovine Diarrhea Virus (BDV) was added to the samples before RNA extraction and also run in a parallel RT-PCR assay. Supernatant from DENV-3 cell cultures was included as an external control. DENV-3 supernatant was previously quantified by a commercial dengue RT-PCR kit (RealArt; artus/QIAGEN, Germany) [15] and used to generate a standard curve. The detection limit of this assay was 100 copies/ml.

### CD4+ and CD8+ T cell counting

Peripheral blood absolute CD4+ and CD8+ T cell counts were assessed using the BD Multitest CD3-FITC/CD8-PE/CD45-PerCP/CD4-APC monoclonal antibody (mAb) cocktail from BD Biosciences (San Diego, CA), according to the manufacturer’s instruction, using a FACSCanto flow cytometer (BD Biosciences). Cell surface staining was routinely performed on 100 μL fresh whole blood.

### Surface immunophenotyping in flow cytometry

Peripheral blood mononuclear cells (PBMCs) were isolated from fresh EDTA-treated blood by Ficoll-Hypaque gradient centrifugation and frozen in liquid nitrogen as previously described [16]. PBMC were isolated from volunteers and stored in liquid nitrogen until used in the assays. To characterize the activation profile of CD8+ T lymphocytes, we used the markers HLA DR and CD38. HLA DR is a transmembrane glycoprotein encoded by genes within the Human Leucocyte Antigen (HLA) complex. CD38 is a nonlineage-restricted type II transmembrane glycoprotein that has emerged as a multifunctional protein. Cells expressing both markers are likely to be activated. The following monoclonal antibodies were used in the FACS assays: anti-CD8-peridin chlorophyll protein (PerCP) (clone SK1), CD45RA-fluorescein isothiocyanate (FITC) (clone L48), CD38-phycoerythrin (PE) (clone HB7), from BD Biosciences (San Jose, CA, USA); CCR7-phycoerythrin—cyanine (PE-Cy7) (clone 3D12), HLADR-Alexa 700 (clone L243), CD27-APCH7 (clone M-T271), CD4-Pacific Blue (clone RAPA-T4), from BD Pharmingen (San Jose, CA, USA); CD3-ECD (clone UCHT1), from Beckman Coulter (Marseille, France) and Fixable Aqua dead cell stain kit, from Molecular Probes (Oregon, USA).

After thawing, cells were centrifuged at 1,500 rpm for 5 minutes and transferred into 96 V bottom well plates (Nunc, Denmark) in 100 L of staining buffer (PBS supplemented with 0.1% sodium azide [Sigma] and 1% FBS, pH 7.4–7.6) with the surface monoclonal antibodies panel. Cells were incubated at 4C in the dark for 30 minutes, washed twice, and resuspended in 100 L of fixation buffer (1% paraformaldehyde Polysciences, Warrington, PA in PBS, (pH 7.4–7.6). Fluorescence minus one (FMO) was used for gating strategy [17]. The strategy is shown in S1 Fig.

### Intracellular staining in flow cytometry

CD8+ T cell proliferation was assessed using a Ki67 staining protocol. Ki67 is a cell-cycle-associated antigen expressed exclusively in proliferating cells. After staining with surface markers CD3-PERCP (clone SK7), CD8-allophycocyanin cyanine-7 (APC-Cy7) (clone SK7), from BD Biosciences (San Jose, CA, USA); CD4-Alexa 700 (clone RAPA-T4), from BD Pharmingen (San Jose, CA, USA) and Fixable Aqua dead cell stain kit, as described above, cells were fixed with 4% fixation buffer for 10 minutes. Cells were washed with staining buffer once and re-suspended in 100 L of permeabilization buffer from BD Biosciences (San Jose, CA, USA) and incubated for 15 minutes. Cells were washed with staining buffer twice. Ki-67-FITC (clone B-56) was added and cells incubated at 4C in darkness for 30 minutes. Finally, the cells were washed twice, and re-suspended in 100 L of 1% fixation buffer.

Samples were acquired on a FACSFortessa, using FACSDiva software (BD Biosciences), and then analyzed with FlowJo software version 9.4 (Tree Star, San Carlo, CA). Fluorescence voltages were determined using matched unstained cells. Compensation was carried out with CompBeads (BD Biosciences) single-stained with. Samples were acquired until at least 200,000 events were collected in a live lymphocyte gate. The analysis strategy is shown in S2 Fig.

### Statistical analysis

Because most continuous variables presented an overdispersed distribution, results were summarized as medians and 25% to 75% interquartile ranges (IQR) and compared across dengue patient groups and non exposed controls, using nonparametric Kruskal-Wallis or Mann-Whitney tests (continuous variables). When the Kruskal-Wallis test indicated a statistically significant difference (*P<*0.05) among more than two groups, a Dunn’s multiple comparison post-tests was carried out to determine between which groups the differences were sustained. Potential correlations were explored using Spearman rank correlation tests. The software Prism, version 5.0, was used for analyses (GraphPad Software, San Diego, CA).

## Results

### Subjects and cell blood counts

Peripheral venous blood was obtained from 74 patients with acute dengue fever and 17 matched donors who were asymptomatic and negative for DENV IgM, NS1, and RNA. The characteristics of the dengue fever patients and healthy controls are depicted in Table 1. No differences were seen in gender and age distribution comparing both groups. As expected, dengue fever patients had lower number of platelets (median 152,000 cells/μl, interquartile range 25%–75% [IQR], 110,000–207,000) when compared to controls (median 226,000 cells/μl, IQR, 166,000–310,000), p<0.0001. Platelets decreased during the first days of disease, with a median of 174,000 cells/μl (IQR, 147,000–232,000) on days 1 and 2, 153,000 cells/μl (IQR, 115,000–206,000) on days 3 and 4, and 94,000 cells/μl (IQR, 28,000–154,000) on days 5 and 6 after the onset of symptoms.

**Table 1:** Demographic characteristics of dengue patients and healthy donors.

Characteristics		DENV infection (n = 74)	Healthy Donors (n = 17)
**Age, in years** (Median and IQR*)			
	Female	44 (31–54)	40 (34–55)
	Male	38 (30–47)	25 (23–31)
**Gender** (%)			
	Female	58	65
	Male	42	35

* Interquartile Range 25%- 75%

Overall leukocyte counts were also lower (median 4,400 cells/μl, IQR, 3,275–6,400) compared to controls (median 8,100 cells/μl, IQR, 6,140–9,335), p<0.0001. Numbers were lower on days 1 and 2 (median 5,100 cells/μl, IQR, 3,750–6,450) and 3 and 4 (median 3,600 cells/μl, IQR, 3,100–5,100), p<0.001, but recovered on days 5 and 6 to levels of the control group.

A subset of 30 dengue fever patients (Table 2) was selected for expanded immunophenotyping experiments. To be representative of the disease natural history after onset of symptoms, 10 of these patients were at days 1 and 2, 10 patients at days 3 and 4, and 10 patients at days 5 and 6, as detailed in Table 2, along with 17 healthy controls.

**Table 2:** Demographic and hematologic characteristics of study participants.

Identification Number	Gender	Days of Symptoms	Age (years)	Dengue Duo NS1/IgM/IgG	DENV* Load (copies/ml)	Platelets (cells/μl)	Lymphocyte count (cells/μl)	T cell count (cells/μl)	CD8+ T cell count (cells/μl)
5	F	6	77	- / + / +	284	10.000	3136	2684	1839
6	M	5	27	- / + / +	87	42.000	1500	665	120
7	F	5	67	- / + / +	84	56.000	6201	4545	1882
14	F	1	35	- / + / +	107	253.000	1779	1315	310
15	F	6	53	- / + / +	141	42.000	4264	3164	1826
26	M	4	46	+ / + / +	11994	237.000	1168	710	375
27	F	4	29	- /—/ -	666	216.000	974	731	139
29	F	1	55	- /—/ -	2840000	113.000	644	411	186
31	F	1	31	+ /—/ -	45839	147.000	840	659	182
32	M	1	26	- /—/ -	59	234.000	2126	1490	666
34	M	3	50	+ /—/ -	14200000	158.000	2236	1623	268
35	F	4	37	- /—/ -	164	149.000	2349	1724	386
37	F	2	46	- /—/ -	896004	132.000	792	602	147
40	F	6	37	- / + / +	0	152.000	1963	1627	542
41	F	3	30	- /—/ -	114	361.000	2141	1674	465
45	M	5	53	- / + / +	0	20.000	2368	1428	416
58	F	2	24	- /—/ +	352	297.000	4132	2835	998
65	M	5	49	+ / + / -	205151	119.000	1190	499	191
66	M	2	30	- / + / +	1983	237.000	1285	1086	443
67	M	3	33	- /—/ -	11828	137.000	986	621	207
69	M	2	56	- /—/ -	20700000	122.000	955	716	298
70	F	3	50	- /—/ -	383	255.000	1680	1211	345
71	M	6	27	+ / + / +	265	237.000	1418	1106	310
98	F	5	22	- /—/ +	858	71.000	2436	1898	970
99	F	3	83	+ /—/ -	1680000	127.000	1605	1445	352
100	F	1	31	- /—/ -	188	222.000	2139	1546	540
102	F	2	26	- /—/ -	7370000	154.000	992	675	152
106	F	4	82	- / + / +	400455	10.000	2442	1702	175
111	F	4	54	+ / + / +	674	95.000	1343	1121	294
138	F	6	70	- / + / +	237	24.000	2419	1340	331

* DENV: dengue virus

### CD8+ T lymphocytes numbers increase late in the course of acute dengue fever

**Fig 1:**
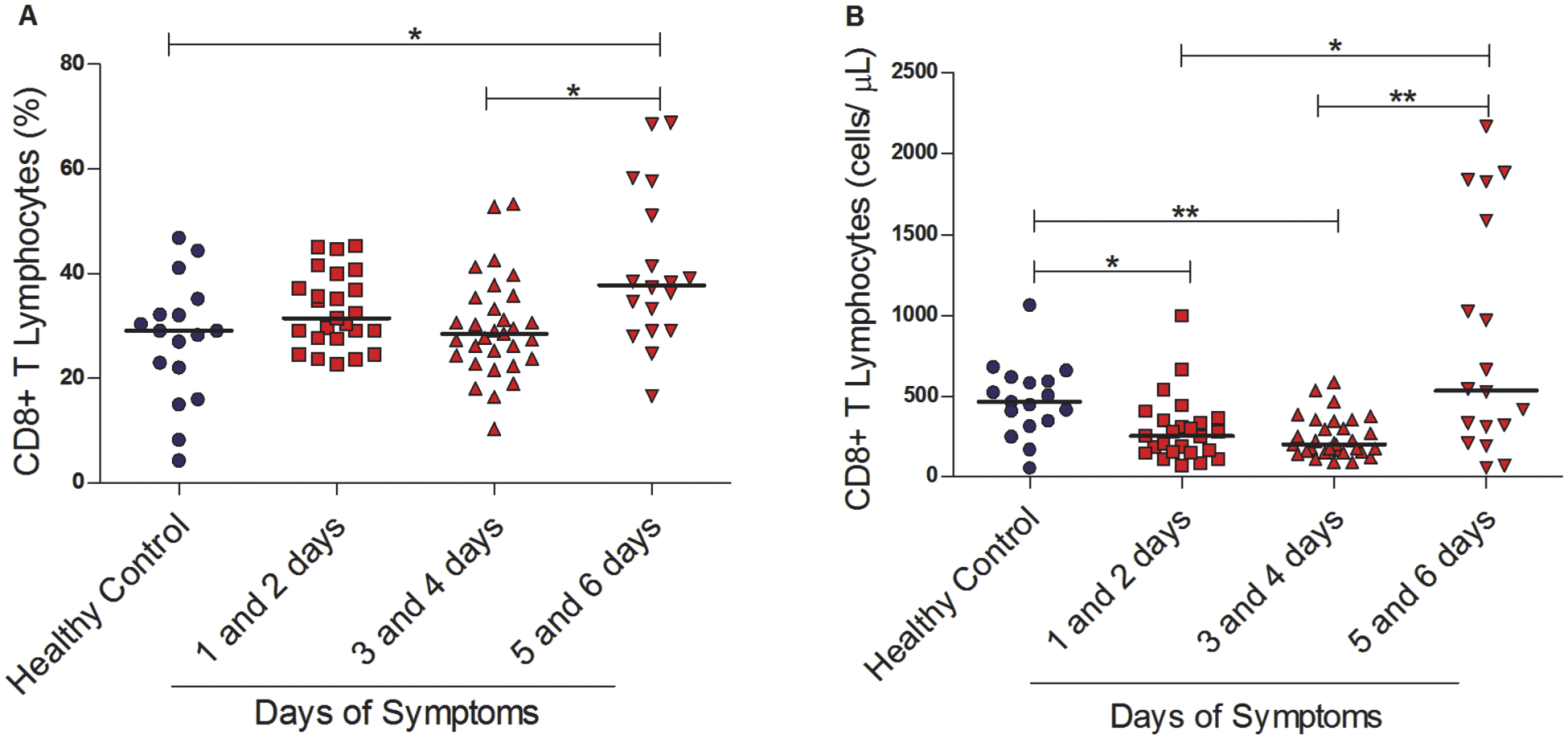
CD8+ T lymphocytes in the course of dengue fever. (A) Percentage within T cells and (B) absolute numbers of CD8+ T lymphocytes in the peripheral blood from healthy controls (n = 17) and dengue fever patients at different days of symptoms (n = 74). A significant change in the percentage of CD8+ T lymphocytes was observed, between days 5 and 6 after the onset of symptoms when compared to healthy controls and days 3 and 4 of symptoms, as shown in A. However, decreased absolute CD8+ T lymphocyte numbers were documented in the first four days of symptoms when compared to healthy controls, returning to higher levels later in the disease’s course, as shown in B. *p< 0.05, ** p<0.01.

We first evaluated the percentages and the absolute numbers of CD8+ T lymphocytes in acute dengue fever patients. As shown in Fig. 1A, the percentage of CD8+ T cells of overall circulating lymphocytes remained constant up to the fourth day after onset of symptoms. However, this percentage increased on days 5 and 6, with an increased median of 38%, IQR, 29–53 (p<0.05), and this was higher than observed in healthy controls.

Absolute numbers of CD8+ T cells were lower from the first to the fourth days after onset of symptoms (median 253 cells/μl, IQR, 151–358 for days 1 and 2; median 201 cells/μl, IQR, 158–345 for days 3 and 4) when comparing dengue patients with healthy controls (median 465 cells/μl, IQR, 329–605). On the fifth and sixth days, we observed a higher number of cells (median 534 cells/μl, IQR, 285–1644), with wider distribution values (Fig. 1B).

### Dengue viral load inversely correlated with the number of CD8+ T cells

**Fig 2:**
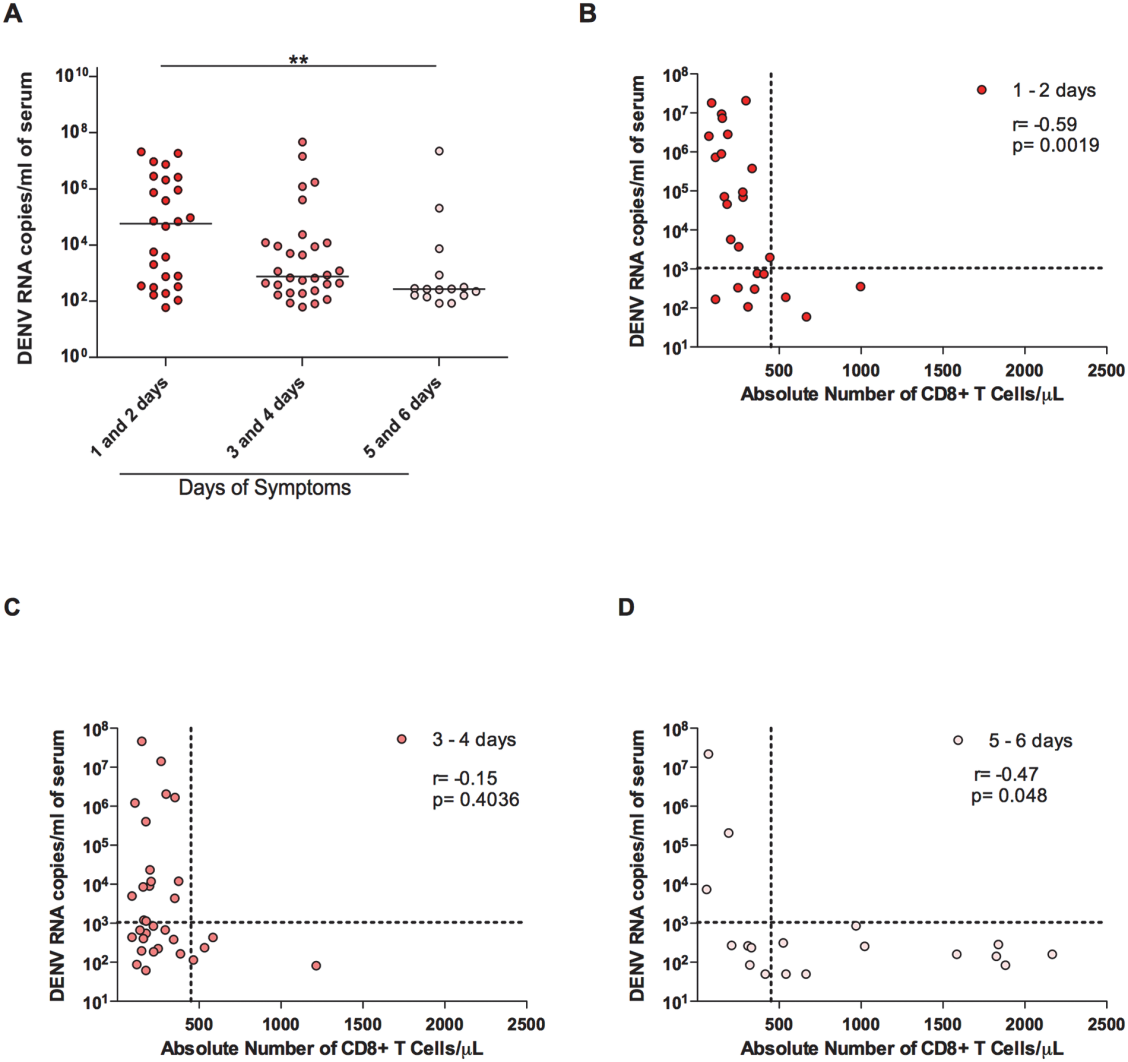
Correlation of CD8+ T lymphocyte numbers and DENV viral load. In A, viral load was determined on different days of symptoms and a significant decrease was observed in days 5 and 6 when compared to days 1 and 2 after the onset of symptoms (p value = 0.0136). In B, a negative correlation was observed between 1 and 2 days of symptoms (r = -0.5900, p = 0.0019). There is no correlation between 3 and 4 days of symptoms (C) and viral loads; In D, a negative correlation was observed between viral loads and 5 and 6 days of symptoms (r = - 0.4705, p = 0.0488). Dashed lines are arbitrarily set at 450 CD8+ T lymphocytes/μL and 1050 copies of RNA/ml.

Dengue viral load was evaluated in the course of dengue fever. As expected, higher viral loads were observed in the first and second days after onset of symptoms, as shown in Fig. 2A, decreasing thereafter. Remarkably, dengue viral load negatively correlated with the number of circulating CD8+ T lymphocytes. As demonstrated in Fig. 2, we observed that higher viral load was seen only when CD8+ T lymphocytes remained below 450 cells/μl (arbitrary dotted line parallel to the y axis), whereas higher CD8+ T lymphocyte counts were associated with Dengue viral load bellow 1,050 copies/ml (arbitrary dotted line parallel to the x axis). These results imply that these CD8+ T cells may be playing a role in the control of DENV replication in the acute phase of disease. Of note, statistically significant correlations (p<0.05) were seen on days 1 and 2 (r = -0.6) (Fig. 2B) and on days 5 and 6 (r = -0.5) (Fig. 2D). In contrast, no correlation was observed on days 3 and 4 (Fig. 2C).

### CD8+ T lymphocytes express Ki67 later in dengue fever

Samples from 30 patients with dengue fever were selected for the remaining experiments. The aim was to be representative of the disease natural history, up to six days after onset of symptoms. Results are shown for days 1 and 2 (10 patients), 3 and 4 (10 patients), and 5 and 6 (10 patients), as detailed in Table 2. These were compared to the 17 healthy controls.

**Fig 3:**
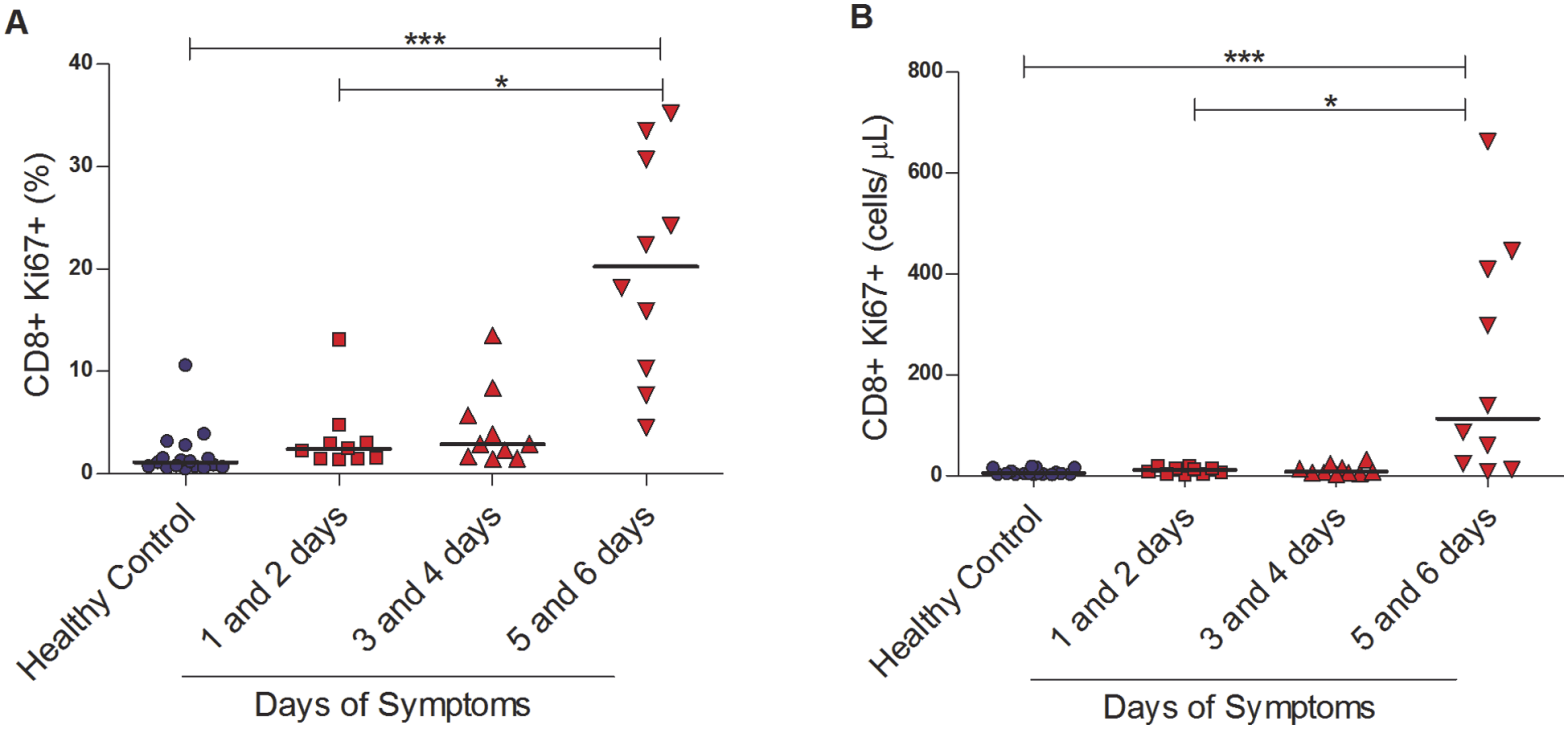
Proliferation of CD8+ T lymphocytes in dengue fever. (A) Percentage and (B) absolute numbers of Ki67+ within CD8+ T lymphocytes in the peripheral blood from healthy controls (n = 17) and dengue fever patients (n = 74) at different days of symptoms. *p<0.05, ***p<0.0001.

We observed that more CD8+ T lymphocytes expressed Ki67 in dengue fever cases when compared to controls, either expressed in absolute numbers or percentage of stained cells (median 14 cells/μl, IQR, 7–40 *vs*. 4 cells/μl, IQR, 3–12, p = 0.002; median 4%, IQR, 2–14 *vs*. median 1%, IQR, 1–2, p<0.0001). However, this increase in expression was largely seen on days 5 and 6 (median 113 cells/μl, IQR, 21–418 and median 20%, IQR, 10–31), suggesting that these cells proliferate later in the course of the disease (Fig. 3).

### Effector memory CD8+ T lymphocytes activate later in the course of dengue fever

**Fig 4:**
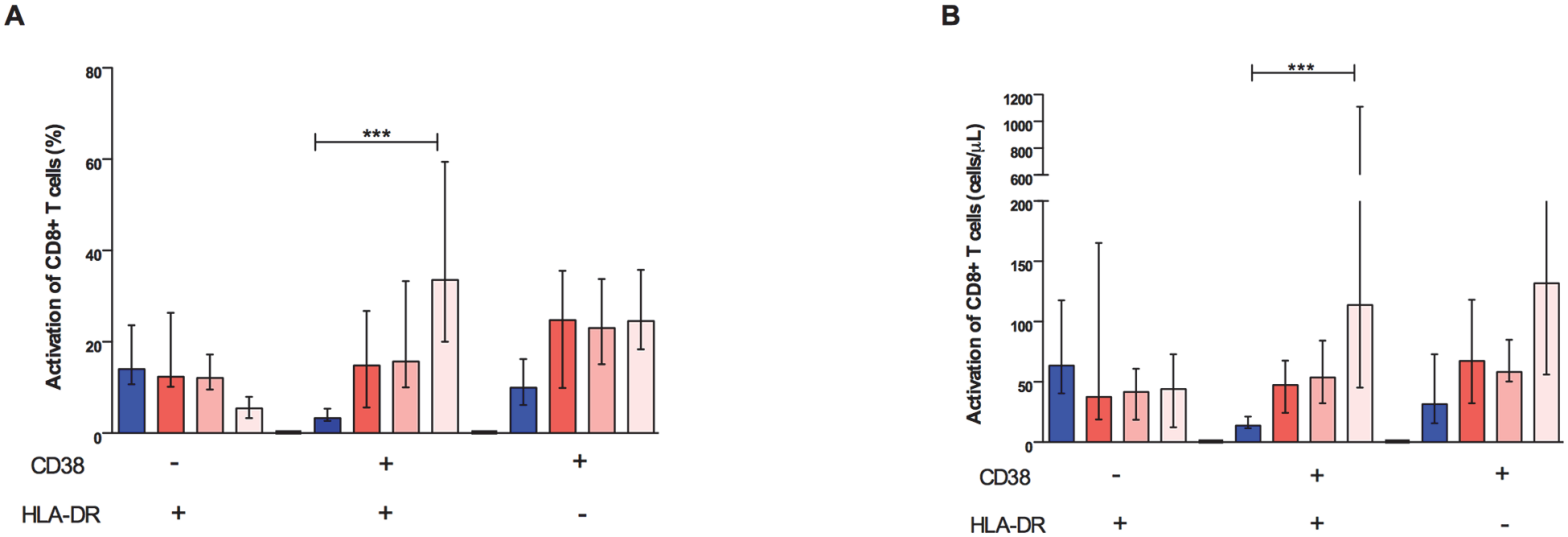
Activation of CD8+ T cells in dengue fever. (A) Percentage and (B) absolute numbers of three subpopulations (HLADR+, CD38+HLADR+, and CD38+) within CD8+ T cells in the peripheral blood from healthy controls (n = 17) and dengue fever patients (n = 74) at different days of symptoms: HLADR+, CD38+HLADR+, and CD38+. Only the dual positive subpopulation significantly increased during the course of dengue fever, either in their percentage or absolute numbers. *p< 0.05, ***p< 0.0001.

We addressed the levels of CD8+ T lymphocytes activation using surface staining for CD38 and HLA-DR. Coinciding with CD8+ T lymphocyte expansion and proliferation, higher cell activation could be detected later in the course of disease, on days 5 and 6, compared to controls either in percentages (median 34%, IQR, 20–59 *vs*. median 3, IQR, 3–5, p<0.0001) or in absolute numbers (median 114 cells/μl, IQR, 45–1110 *vs*. median 14 cells/μl, IQR, 12–21, p<0.0001), as depicted in Fig. 4.

**Fig 5:**
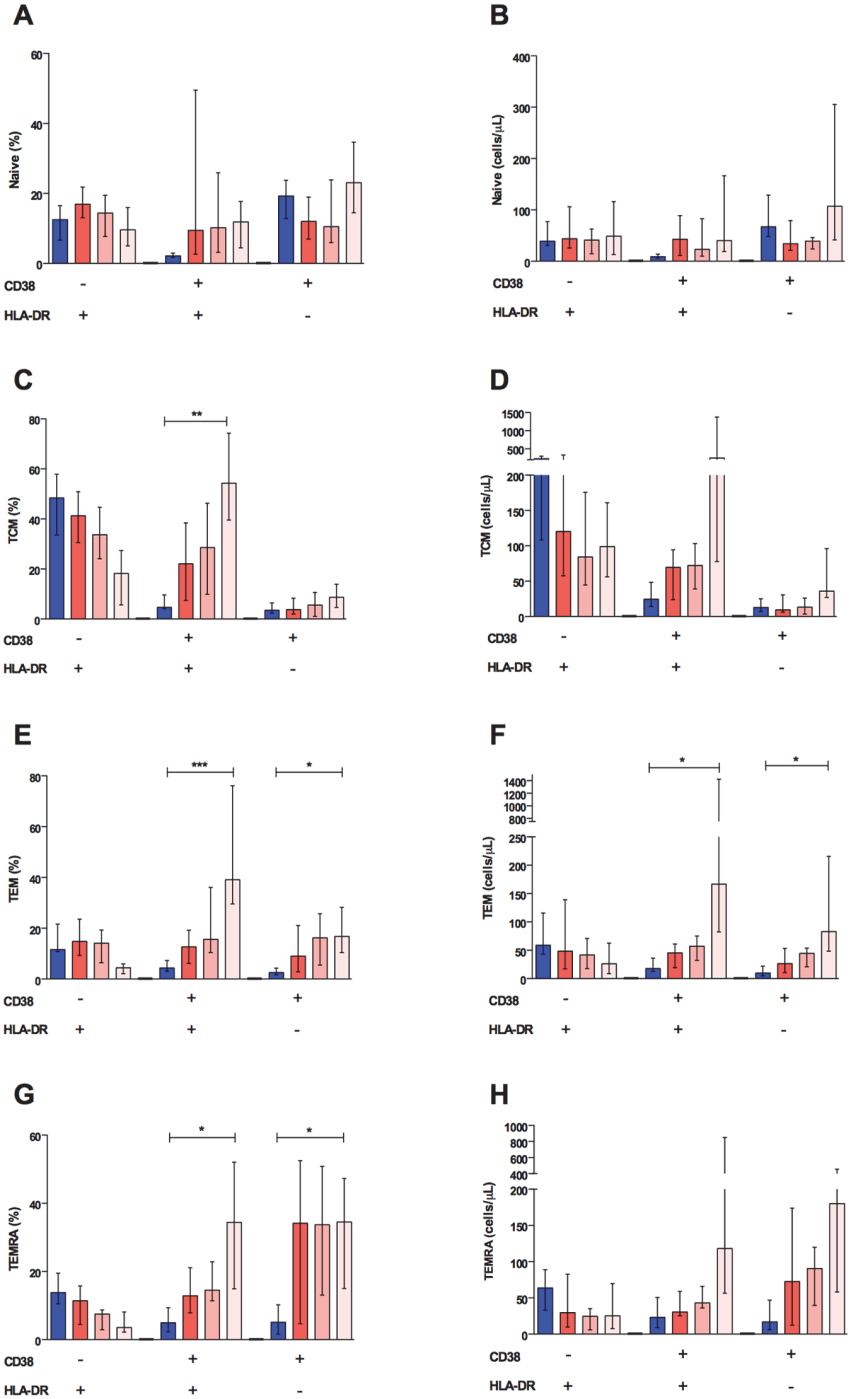
Activation of CD8+ T cells subpopulations in dengue fever. Percentage and absolute numbers of naïve (A and B), central memory (C and D), effector memory (E and F), and terminal effector memory (G and H) T CD8+ cells are shown. Increased percentages of activated cells were observed mostly in dual positive CD38+HLA-DR+ central memory (TCM) (p<0.001) effector memory (TEM) (p<0.0001) and terminal effector memory (TEMRA) (p<0.01) T cells, although the percentage of CD38+HLA-DR- effector memory (p<0.01) and terminal effector (p<0.01) T cells were also augmented in dengue fever.

The cellular activation profile was different among the subpopulations of CD8+ T lymphocytes. Using comprehensive staining panels, we did not observe statistically significant differences in activation of naïve cells (Fig. 5A and 5B). On the other hand, higher activation was observed on days 5 and 6 in the central memory (TCM), effector memory (TEM), and terminally effector memory (TEMRA) cell percentages (Fig. 5C, 5E, and 5G, respectively). Nevertheless, this effect was only seen in the TEM subpopulation when absolute numbers were evaluated (Fig. 5F), suggesting that TEM cells are largely responsible for this phenomenon. We also observed that the TEM CD8+ T cell subset was negatively correlated with DENV viral load, suggesting that the activation of such particular phenotype may have a central role in controlling virus replication; however, given the post-hoc nature of this analysis this result needs to be interpreted with caution, requiring confirmatory experiments.

## Discussion

Changes in lymphocyte subsets in dengue fever have long been recognized, including an increase in CD8+ T lymphocyte numbers [8,18,19]. Using samples collected in a DENV2 outbreak in the coast of the State of São Paulo, Brazil [12], we were able to demonstrate that the percentage CD8+ T cell count increased later in the course of disease, after onset of symptoms. This was associated with higher Ki67 expression, suggesting a proliferative rebound that follows the peak viremia. This phenomenon may be related to increased cell activation, as has been suggested by others [20]. In this paper we explored in more detail the activation status of different CD8+ T cell subpopulations in adult patients with dengue fever.

During high viral burden, several circulating cells are activated including monocytes [21], NK cells, CD4+ and CD8+ T cells [8,22,23]. This activation seems to be a natural immune response to the pathogen and reflects its efforts to control viral replication. Our results show that the expanding number of CD8+ T cells was associated with lower viremia, especially later in the disease course. Dung *et al*., also demonstrated that activated CD8+ T cell expansion (evaluated by the expression of HLA-DR and CD38 molecules) was associated with viral control [24]. However, it is possible that high levels of cellular activation may be harmful to the host and may be related to disease severity. A number of studies have found increased markers of immune cell activation in patients with dengue hemorrhagic fever (DHF) compared with patients with classic dengue fever (DF) [25]. Indeed, children who developed DHF had higher percentages of CD8+ T cells and NK cells expressing CD69, an early activation marker than those with DF during the febrile period of illness [8,26]. Also, children admitted with acute dengue fever had increased levels of NK cells and T lymphocyte activation and the severity of disease was associated with higher activation status [23].

In the last few years considerable progress has been made in identifying different T cell memory subsets to dissect the heterogeneity of human immune responses [27]. In this paper, we evaluated in different CD8+ T subpopulations in adults with dengue fever using a comprehensive panel of antibodies. Our current study demonstrates differences in activation status among the various CD8+ T cell subpopulations in dengue fever patients. The percentages and numbers of effector memory (TEM) cells, characterized by the CCR7-CD27+CD45RA+/- phenotype [28], were the most activated in the later phase of the disease, as demonstrated by the expression of HLADR and CD38 molecules. Other subpopulations also exhibited increased activation, including central memory (TCM) and terminally differentiated memory cells (TEMRA). However this finding was restricted to the percentage and not the absolute numbers of these two TCM and TEMRA subsets suggesting that TEM cells are the most activated subset in the later stages of acute dengue fever. TEM cells have immediate effector function, by secreting IL-2, IFNγ, and other cytokines in response to infectious pathogens [29,30,31].

One limitation of our study is the lack of any data regarding antigen-specific responses since the observed expansion of TEM cells was described in CD8+ T cells using only surface staining. Further studies using DENV-derived proteins or peptides for stimulation of PBMC from acute dengue cases are warranted. Antigen-specific CD8+ T cell responses have been recently described in general population from Sri Lanka hyperendemic area, with higher magnitude and more polyfunctional responses for HLA alleles associated with decreased susceptibility to severe disease [32].

DENV-reactive CD8+ T cells are important in the control of viral replication [33] and may have different responses to different epitopes [34]. DENV serotype-cross-reactivity of CD8+ T cells has also been demonstrated after primary infection [35]. The observed expansion of TEM cells, which may contain such cells, should be explored in future studies to verify their antigen-specific characteristics [24]. The analysis of human memory T and B cells has the capacity to identify the antigens that are targeted by effector T cells, thus providing a rational for vaccine design. In fact, many dengue vaccine candidates have been using replicating virus, including chimeric dengue virus [36], which can induce a significant immune reaction against the vaccine [37] [38] [39].

Based on our findings that different CD8+ T cell subpopulation are activated to different levels, it may be important to investigate the status of CD8+ T cell differentiation when analyzing antigen-specific responses. Considering the key role of CD8+ T cell activation and antigen-specific responses in the pathogenesis of dengue fever, further investigation should be conducted to explore the mechanisms of activation pathways in disease pathogenesis.

## Supporting Information

S1 Fig Analytical strategy to assess the activation of naïve and memory CD8+ T cells. A gate on single cells was determined by the size in relation to height (FSC-H) and relative area (FSC-A). Then a gate was made in the total lymphocyte population followed by a gate on the CD3+ population. Thereafter, two other gates were made in populations of T lymphocytes (CD4+ and CD8+). Within the population of CD8+ T lymphocyte subpopulations were evaluated naïve and memory from the Boolean analysis, which was made all possible combinations with the markers CCR7, CD45RA, CD27. Within each population (naïve, TCM, TEM and TEMRA) a quadrant gate was carried out to evaluate the expression of activation markers HLA-DR and CD38.
(TIF)

S2 Fig Analytical strategy to assess the expression of Ki67 in CD8+ T cells. A gate on single cells was determined using forward scatter height (FSC-H) and relative area (FSC-A). The lymphocyte population was delimited followed by a gate on CD3+ cells. CD8+ T cells were identified within the CD3+ T cells and analyzed for Ki67 expression.
(TIF)
