# Supplementary figures and images for: CD8+ T Lymphocyte Expansion, Proliferation and Activation in Dengue Fever
Source: PLoS Negl Trop Dis. 2015 Feb 12;9(2):e0003520. doi: 10.1371/journal.pntd.0003520 (PMC4326415; doi:10.1371/journal.pntd.0003520)

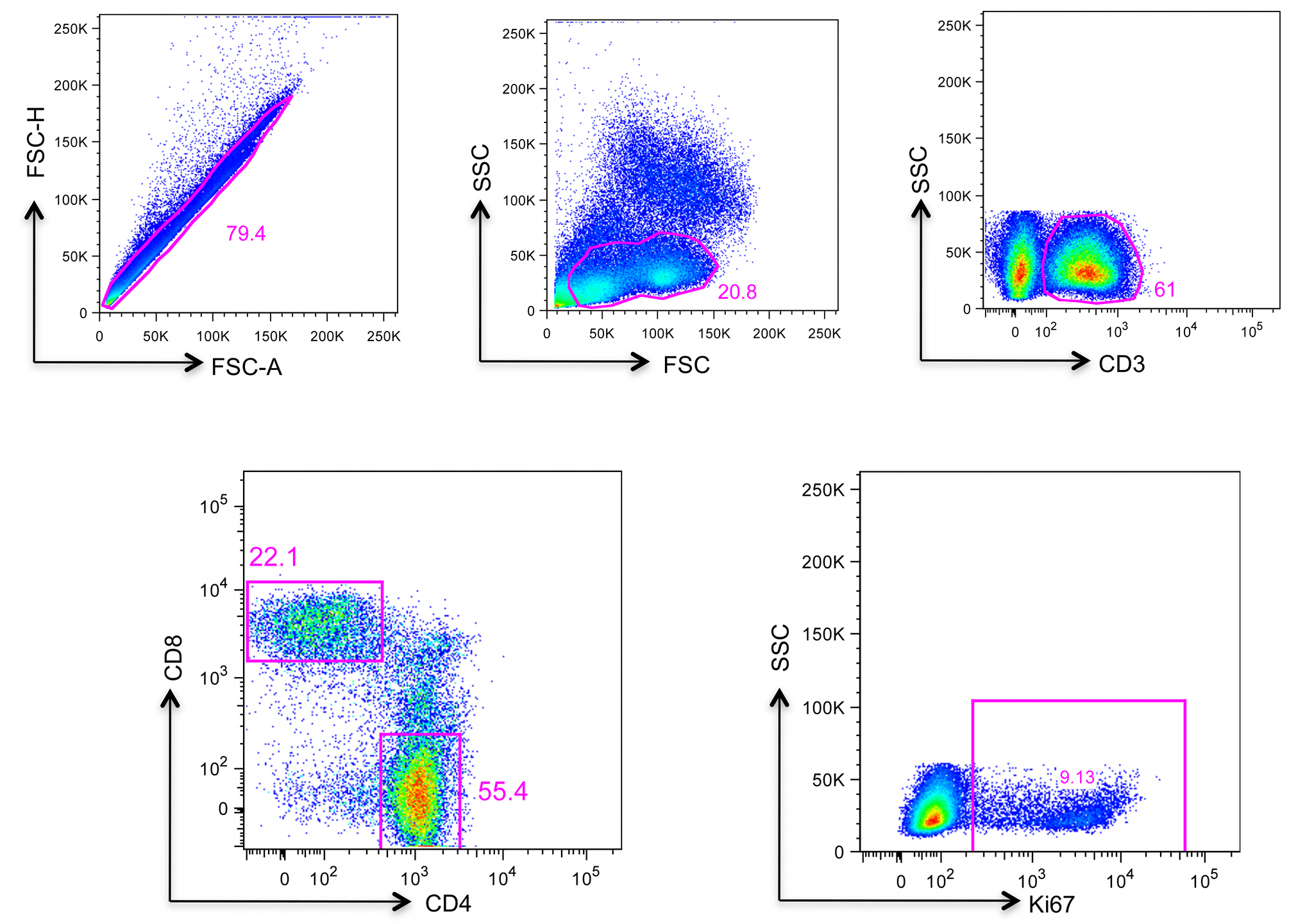

Supplement: S1 Fig — A gate on single cells was determined by the size in relation to height (FSC-H) and relative area (FSC-A). Then a gate was made in the total lymphocyte population followed by a gate on the CD3+ population. Thereafter, two other gates were made in populations of T lymphocytes (CD4+ and CD8+). Within the population of CD8+ T lymphocyte subpopulations were evaluated naïve and memory from the Boolean analysis, which was made all possible combinations with the markers CCR7, CD45RA, CD27. Within each population (naïve, TCM, TEM and TEMRA) a quadrant gate was carried out to evaluate the expression of activation markers HLA-DR and CD38. (TIF) [file pntd.0003520.s001.tif]

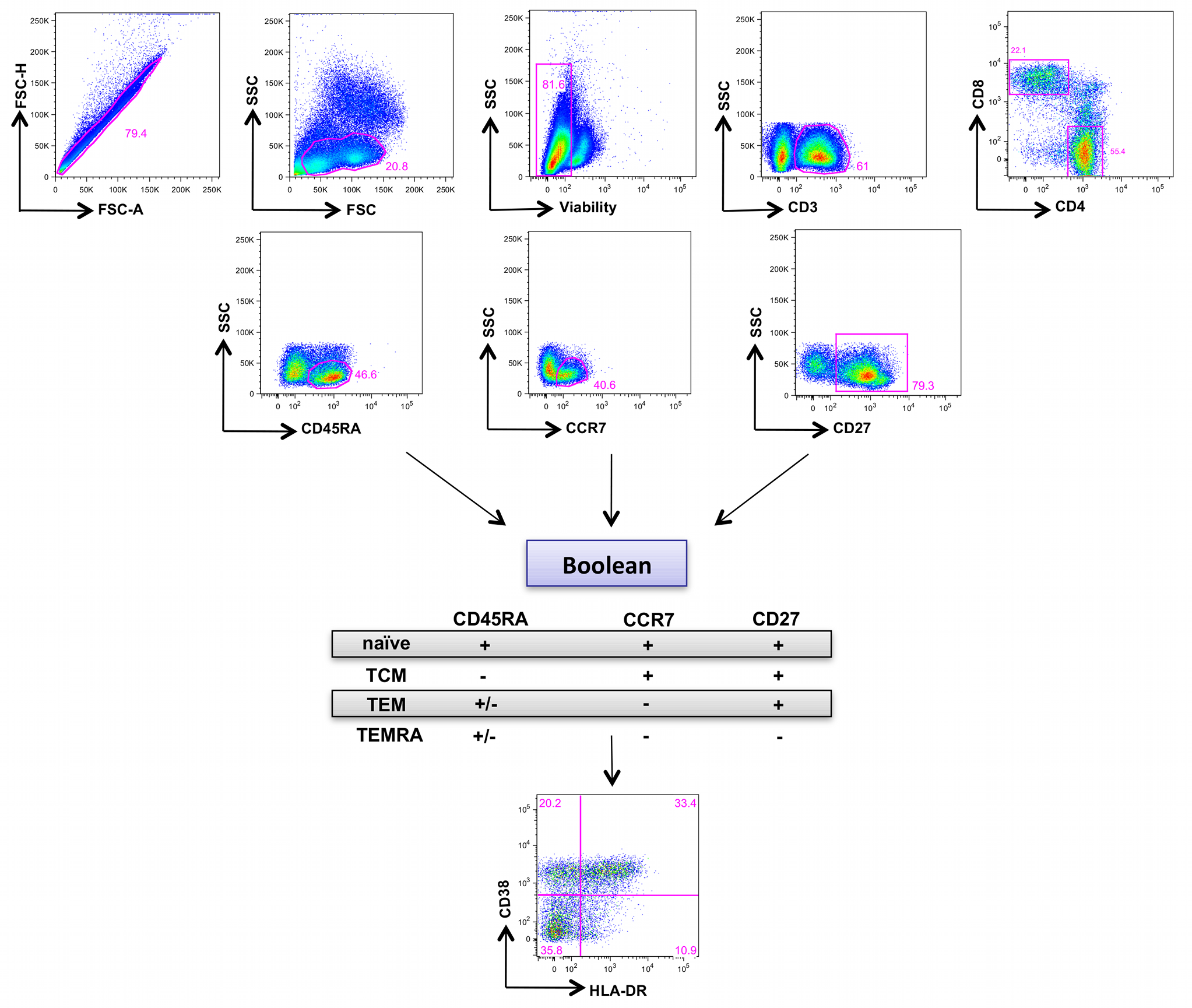

Supplement: S2 Fig — A gate on single cells was determined using forward scatter height (FSC-H) and relative area (FSC-A). The lymphocyte population was delimited followed by a gate on CD3+ cells. CD8+ T cells were identified within the CD3+ T cells and analyzed for Ki67 expression. (TIF) [file pntd.0003520.s002.tif]
